# GC-MS Analysis of Methadone and EDDP in Addicted Patients under Methadone Substitution Treatment: Comparison of Urine and Plasma as Biological Samples

**DOI:** 10.3390/molecules27238360

**Published:** 2022-11-30

**Authors:** Daniela-Mădălina Ciucă Anghel, Anne-Marie Ciobanu, Claudia Maria Guțu, Miriana Stan, Gheorghe Tudor, Daniela Luiza Baconi

**Affiliations:** 1 Department of Toxicology, Carol Davila University of Medicine and Pharmacy, 37 Dionisie Lupu Street, Sector 2, 20021 Bucharest, Romania; 2 Department of Medicines Control, Carol Davila University of Medicine and Pharmacy, 37 Dionisie Lupu Street, Sector 2, 20021 Bucharest, Romania; 3 Department of Systematic Theology and Sacred Art, Orthodox Theology—Social Work Section, “Justinian the Patriarch” University of Theology, 29 Berzei Street, Sector 1, 010521 Bucharest, Romania

**Keywords:** methadone, substitution treatment, EDDP, liquid–liquid extraction, GC-MS, diphenylamine

## Abstract

(1) Background: Methadone, along with buprenorphine, is the most commonly used drug for the treatment of opioid dependence. This study aimed to analyze methadone and its major metabolite, 2-ethylidene-1,5-dimethyl-3,3-diphenyl pyrrolidine (EDDP), in the urine and plasma of opiate addicts. The study group consisted of drug users voluntarily admitted to the detoxification center C.E.T.T.T. “St. Stelian” of Bucharest. Secondly, the study aimed to identify whether urine or plasma provides better results for the proposed method. (2) Methods: A GC-MS method, using an internal standard (diphenylamine) in the FULL-SCAN and SIM modes of operation and using the m/z = 72 ion for methadone and the m/z = 277 ion for EDDP, combined with a liquid–liquid extraction procedure was performed. (3) Results: The applied procedure allows the detection and quantification of methadone in both urine and plasma samples. EDDP was identified in patients with higher levels of methadone. Higher levels of methadone were detected in urine than in plasma samples. (4) Conclusions: This procedure can be used in clinical laboratories for the rapid determination of methadone levels in urine rather than in plasma. The procedure can be applied for the monitoring of methadone substitution treatment.

## 1. Introduction

It is well known that methadone, along with buprenorphine, is the most used drug for the treatment of opioid dependence [1,2]. The dosage of methadone used in maintenance therapy varies, depending on the intensity of withdrawal symptoms. In opioid use disorder, the optimal daily methadone dose is 80 to 150 mg [2]. Studies have shown that although usual doses are approximately between 90 and 100 mg/day, moderate doses of methadone are preferred to successfully prevent abstinence syndrome [1,3,4]. Even though addicts are included in methadone maintenance treatment (MMT) programs, they may either increase the dose or continue to use illicit drugs [5,6]. Methadone has high oral bioavailability (70–95% [7]), and plasma levels are measurable after 30 min. After that, a decline in plasma levels occurs after 24 h [2,8]. Studies have shown a significant correlation between plasma levels of methadone and methadone doses [9]. Variations in methadone blood levels can be explained by inter-individual variations due to polymorphisms [10]. Variability in methadone pharmacodynamics has also been observed [11]. Considering its pharmacokinetics, methadone is metabolized to inactive pyrrolidine and pyrroline metabolites, with its major metabolite being 2-ethylidene-1,5-dimethyl-3,3-diphenyl pyrrolidine (EDDP) [6,8].

A short overview of some methods for the determination of methadone and EDDP in biological samples is presented in Table 1 below. As extraction methods, both SPE (solid-phase extraction) and LLE (liquid–liquid extraction) are outlined. As biological samples, the methods described include blood (in its various forms), hair, and skin but also urine, and both methadone (MTD) and EDDP were quantified.

Undoubtedly, the most frequently used instrumentation for the detection of methadone and EDDP in biological samples is based on chromatography (either liquid or gas) combined with spectrometry (Table 1).

Although LC-MS is still carried out for many analyses, GC-MS remains an important technique applicable for various volatile compounds (flavorings), unidentified residual solvents, fatty acids, anabolic steroids, and drugs of abuse. GC-MS has high applicability in the forensic field, as it allows the characterization of many narcotics due to its high selectivity [18].

The major advantage of the coupled GC-MS method is that electron impact (EI) spectra are obtained. Due to the absorbed energy, the ionization and fragmentation of the molecule are possible. The characteristic and reproducible pathway of the ionization and fragmentation of analytes led to library spectra that can be correlated with the results obtained in the analyses. In addition, the method has the advantage of operating in either full-scan mode (which allows the collection of ions within a given mass range) or selected-ion monitoring (SIM) mode (which allows the collection of only pre-selected masses that are characteristic of the interest analyte). The method has high selectivity, as follows: 1–10 ng for the full-scan method and 1–10 pg for SIM mode (due to a dramatic decrease in background noise). In addition, the method provides an excellent linear range (five or six orders of magnitude). Using a capillary column prevents the loss of vacuum in the mass spectrometer, leading to short retention times and, therefore, to the short time of the analysis. Another advantage of the GC-MS method is the simplicity of the interface, with no special designs being required [18]. Due to its accessibility and lower cost, the method is more feasible than LC-MS.

Considering the pharmacokinetics of methadone and the methods of identification and quantification, we aimed to analyze methadone (identification and dosage) and its major metabolite, 2-ethylidene-1,5-dimethyl-3,3-diphenyl-pyrrolidine (EDDP, identification), in the urine and plasma of opiate addicts. A previously validated GC-MS method [17] combined with LLE sample preparation [9] was performed. Possible relationships between methadone levels in biological samples and methadone doses were investigated. Secondly, given the fact that the most commonly used biological samples are urine and plasma, the study aimed to identify which of them is more appropriate for the proposed method.

## 2. Results

### 2.1. Characterization of the Study Group

A detailed characterization of the study group, including socio-demographic characteristics, history of drug use, methadone maintenance treatment history, and associated medication, is presented in Table 2.

Out of the total of 14 patients included in the study, the majority were men (12 patients, which represents 85.71%) and a small number were women (2 patients, representing 14.29%), with the ratio of men to women being 6.

The mean age of the group was 32.23 ± 9.90 years (32.73 ± 10.71 years for males and 29.5 ± 3.54 years for females) and ranged from 19 to 47 years for the group (19–47 years for men and 27–32 years for women). The most common age group was 20–30 (42.86%). According to medical records, 78.57% of the patients had a history of drug use (opiates), and 50% of them had already been admitted for MMT. For the patients included in the MMT, the mean dose for methadone treatment was 95.71 ± 20.70 mg/day.

Regarding the associated treatment, there have been four classes of medication identified: antidepressants (AD), anxiolytics/hypnotics (AX), antipsychotics (AP), and anticonvulsants (AC), with the most frequent combination being methadone with all four classes of drugs (Table 2).

### 2.2. Qualitative and Quantitative Analyses: Determination of Methadone and Comparison between Urine and Plasma Samples

The biological samples obtained from the patients were analyzed using the GC-MS method described below.

**Figure 1:**
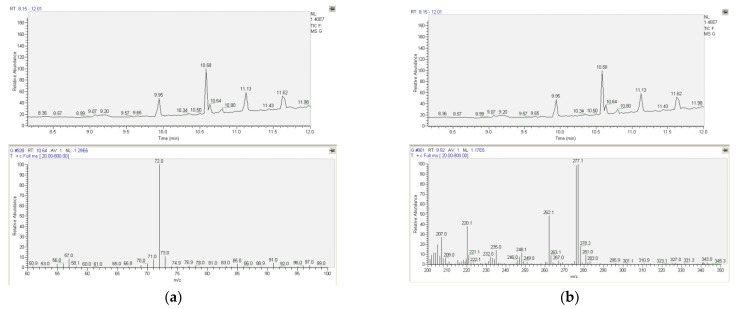
Typical chromatograms and MS spectra for methadone– and EDDP–positive urine samples: (**a**) methadone identification in patients’ urine sample—FULL scan (t_R_ = 10.64, m/z = 72), Patient 7; (**b**) EDDP identification in patients’ urine sample—FULL scan (t_R_ = 9.92, m/z = 277), Patient 7.

**Figure 2:**
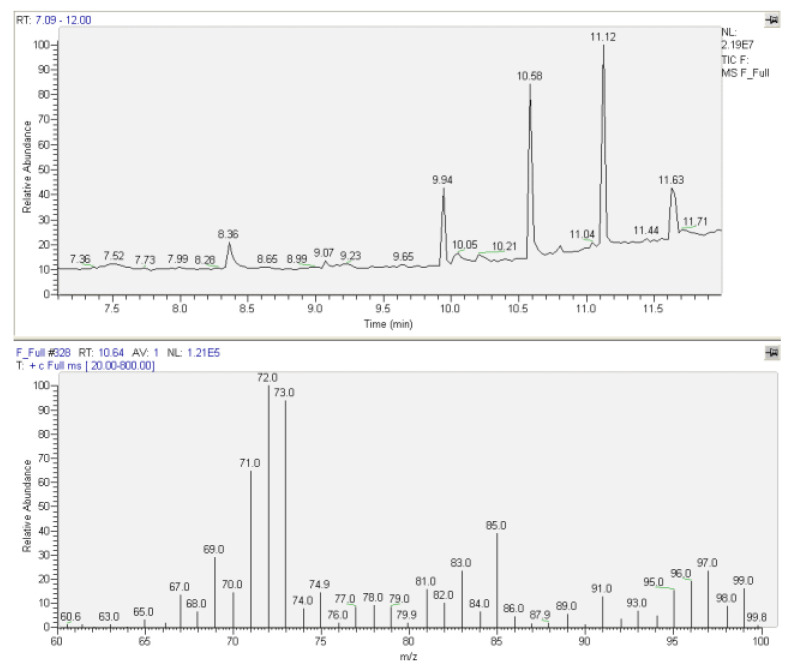
Typical chromatogram and MS spectrum for methadone identification in patients’ plasma sample—FULL (t_R_ = 10.64, m/z = 72), Patient 14.

For qualitative analysis, a FULL-scan analysis under the above-mentioned GC-MS conditions for the identification of both EDDP and methadone in urine and plasma samples based on the retention time (for methadone t_R_ = 10.64 and for EDDP t_R_ = 9.92) but also based on comparing the mass spectra with the spectral library was performed. For the internal standard (diphenylamine), the retention time was 5.93 (m/z = 169, 168, 167). Positive results for methadone detection were obtained in urine samples (patient nos. 4, 5, 6, and 7). For EDDP, positive results were obtained in urine samples for patient nos. 5, 6, and 7. Typical chromatograms and MS spectra for methadone- and EDDP-positive urine samples are presented in Figure 1a,b. For plasma analysis, positive results were obtained for methadone in patient nos. 2 and 14. Typical chromatograms and MS spectra for methadone determination in patients’ plasma samples are presented in Figure 2. For EDDP detection in plasma, no positive results were obtained.

**Figure 3:**
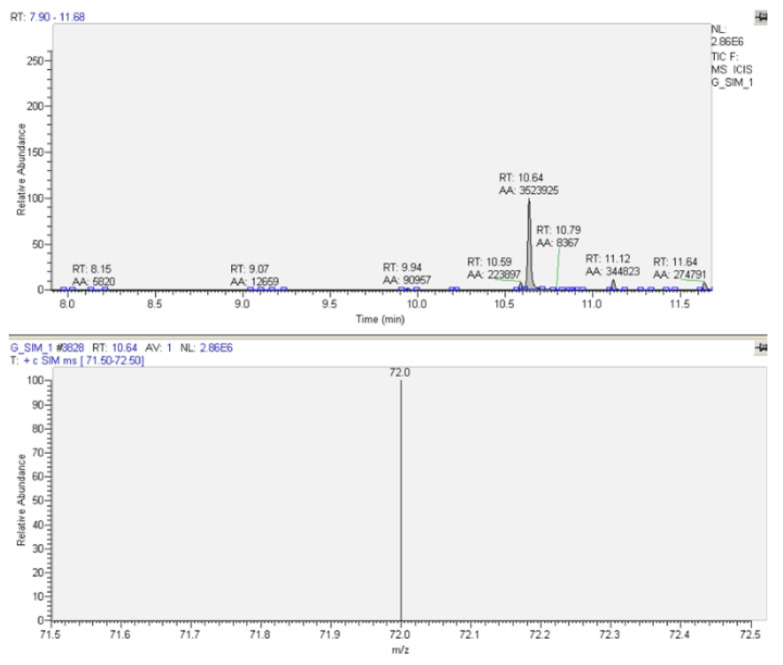
Typical chromatogram and MS spectrum for methadone determination in patients’ urine sample—SIM (t_R_ = 10.64, m/z = 72), Patient 7.

**Figure 4:**
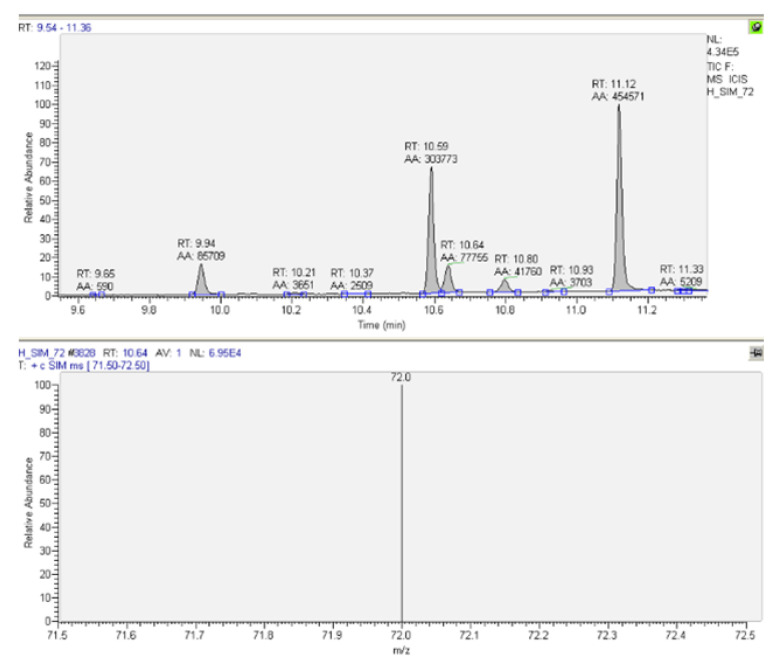
Typical chromatogram and MS spectrum for methadone determination in patients’ plasma sample—SIM (t_R_ = 10.64, m/z = 72), Patient 2.

For the quantitative determination, the samples were also analyzed in SIM mode (selected-ion monitoring), following the ion with m/z = 72 for methadone. A typical chromatogram and MS spectrum for methadone quantification in patients’ urine samples are presented in Figure 3. A typical chromatogram and MS spectrum for methadone quantification in patients’ plasma samples are presented in Figure 4.

The urine and plasma levels of methadone were calculated. For patient 4, the methadone level was 1.06 µg/mL, while for the other three positive patients, the concentration levels exceeded the upper concentration in the calibration curve, and dilution was performed. The methadone concentrations in urine for these three patients were 4.366 µg/mL, 3.012 µg/mL, and 6.697 µg/mL. For plasma, the levels of methadone were 0.072 µg/mL and 0.013 µg/mL for patient 2 and patient 14, respectively (Table 3).

## 3. Discussion

### 3.1. Characterization of the Study Group

Following the characterization of the group, we can outline the prevalence of males in the study group (Table 2). Regarding the distribution by sex and mean age (32.23 ± 9.90 years), there are slightly statistically significant differences between males (32.73 ± 10.71 years) and females (and 29.5 ± 3.54 years). The most common age group is 20–30 (42.86%), highlighting that the onset of heroin use usually takes place during youth. The fact that 78.57% of the patients have a history of drug use (opiates) can be explained by the specificity of the treatment center. In addition, the majority of the patients have an admission diagnosis of opiate dependence, also explained by the specificity of the center.

The high percentage of patients who have already been admitted for methadone maintenance treatment (50%, Table 2) can be explained by the results of the correlation Pearson test (R = 0.8822, the *p*-value is 0.00003) between age and a history of MMT. This indicates a high positive correlation between the parameters (age and history of MMT) with a very high statistical significance. So, the chances of previous MMT increase with age. Regarding the mean dose for methadone treatment, the result in this study (95.71 ± 20.70 mg/day, Table 2) is confirmed by other studies’ results [1,2]. Regarding the associated treatment, in 37.5% of cases, anticonvulsant drugs were associated, and anxiolytics and antipsychotics were equally used (18.75%), while 25% of the patients were under treatment with an antidepressant. These results are consistent with new findings in the field showing a high rate of psychiatric comorbidities in opioid use disorder [19].

### 3.2. Qualitative and Quantitative Analyses: Determination of Methadone and Comparison between Urine and Plasma Samples

Regarding qualitative analysis, the FULL-scan procedure led to positive results for methadone in both urine and plasma samples in almost all patients who had methadone in their treatment.

In urine, using the SIM procedure, the quantitative analysis with diphenylamine as an internal standard revealed concentrations of methadone of 1.06 µg/mL, 4.366 µg/mL, 3.012 µg/mL, and 6.697 µg/mL. We observed that higher levels of methadone in urine could be associated with higher oral doses of methadone (a result supported by other studies [20]), although in one case (patient 5), there was a higher level of methadone in urine than in patient 4, who had the same oral methadone dose (100 mg). This could be explained by either different sampling times, individual variability in the pharmacokinetics of methadone (metabolism and elimination phases), or possible supplementary ingestion of methadone in patient 5. The difference among methadone doses used in patient numbers 4, 5, 6, and 7 is only 10 mg, but the difference in their urinary levels of methadone is significant. We conclude that all of the above-mentioned reasons could be possible.

For EDDP, only the qualitative analysis was performed, using both FULL-scan and SIM procedures. For the patient with a lower level of methadone in urine (patient no. 4), the presence of EDDP was not identified. For patients with higher levels of methadone in urine (>3 µg/mL), the presence of the EDDP metabolite was detected. This result is confirmed by other studies that outlined the relationship between the daily methadone dose and urinary levels of methadone and EDDP [21]. This outcome could also be explained by the possible slower metabolism of methadone in patient 4. In addition, another explanation could be that the continuous administration of methadone causes the induction of methadone metabolism, which results in increased levels of EDDP in urine [15].

Regarding the plasma samples (Table 3), FULL-scan and SIM procedures were performed, and the results were positive for two of the patients who were recorded on methadone treatment (patient 2—100 mg MTD/day; patient 14—100 mg MTD/day). For patient nos. 2 and 14, the methadone levels in plasma were 0.072 µg/mL and 0.013 µg/mL, respectively, while for patient number 12 (50 mg MTD/daily), the result was negative. The results for plasma detection compared to urine detection could be explained either by fluctuation in the plasma concentration from day to day due to tolerance, as studies have recently shown [18,21], or by the fact that the recovery rates of methadone from plasma are smaller. In addition, this result is supported by studies that showed a relationship between the daily dose of methadone and the methadone plasma level [9].

The comparison between methadone levels in urine and plasma reveals higher values for urine samples (Table 3). This result is consistent with studies on the urinary excretion of methadone and the literature data indicating a wide distribution of methadone in urine [7,20].

## 4. Materials and Methods

### 4.1. Study Group

The study group consisted of 14 drug users who were voluntarily admitted to the detoxification center C.E.T.T.T. “St. Stelian” of Bucharest, either at their first admission to the center or having a prior history in the Toxicology Section of the Center. The patients had various diagnoses at admission, such as organic personality disorder (code F07.0), mental and behavioral disorders due to drug use (code F11.2), personal history of drug use disorder (Z86.42), or mood disorder (affective), unspecified (Code F34.9), and the majority of the patients were also diagnosed with heroin addiction, according to ICD-11 (64.28%, 9/14). Data collection from the patients included in the study was performed under the approval of the Ethics Committee of the Center (issue no. 2/15.11.2021) and was carried out in accordance with the Declaration of Helsinki. Informed consent was obtained from all subjects involved in the study.

The medical records included diagnosis at admission, admission symptoms, and psychiatric examination. Socio-demographic data such as sex and age were also collected. Parameters such as the existence of drug use history, the existence of methadone maintenance treatment history, and the methadone dose for treatment were also taken into account. Moreover, biological samples were obtained, including plasma and urine samples in order to reveal a possible correlation between the methadone dose and the methadone level in plasma and urine.

### 4.2. Statistical Analysis

The statistical analysis was performed with EXCEL. Data for various parameters such as age and methadone dose are presented as mean ± standard deviation (SD) (Table 2). In addition, a correlation between age and a history of methadone maintenance treatment was evaluated using Pearson’s correlation. The test was statistically significant at a *p*-value < 0.05.

### 4.3. Biological Samples

Both urine and blood were collected from the patients. The urine samples were directly stored in the freezer, at −20 °C. The blood samples were collected in EDTA (ethylenediaminetetraacetic acid) vacutainers, centrifuged, and separated in order to obtain plasma. The samples of plasma were also stored in the freezer at −20 °C until the analysis.

### 4.4. Chemicals

The methadone (MTD) standard was obtained from Sicomed S.A., Bucharest, Romania. Diphenylamine, used as an internal standard (IS), was obtained from Merck Schuchardt OHG, Hohenbrunn, Germany. Potassium hydroxide, used for the preparation of a 2M solution, was obtained from Lach-Ner, Czech Republic; n-hexane and 2-propanol, used as solvents for extraction, were obtained from Merck Schuchardt OHG, Hohenbrunn, Germany, and Sigma-Aldrich Chemie, Steinheim, Germany respectively. Methanol, used as a solvent for the IS preparation, was obtained from Chemical Company, Iasi, Romania.

The blank urine used for the calibration curve was obtained from a volunteer non-drug user after his informed consent had been obtained.

### 4.5. Preparation of Standard Solution

The standard stock solution of 1 mg/mL methadone was diluted 1:100 in methanol, and the obtained methadone solution (10 µg/mL) was used to prepare calibration standards in blank urine and blank plasma within the range of 0.025–3 µg/mL and 0.01–1 µg/mL, respectively. Diphenylamine 0.01% in methanol was used as IS. The LLE procedure was applied, and the samples were analyzed through the GC-MS procedure.

### 4.6. Liquid–liquid Extraction Procedure

An LLE procedure for methadone was performed using a mixture of n-hexane–2-propanol 97:3 (*v*/*v*) at basic pH.

The procedure of extraction from plasma: To 1 mL of plasma sample, 250 µL of diphenylamine 0.01% in methanol was added. The samples were alkalinized using 2M kalium hydroxide (up to pH 10). Four milliliters of the solvent mixture (n-hexane–2-propanol, 97:3) was added and then vortexed for 15 min, and then the samples were centrifuged for 10 min at 3400 rpm and 15 °C. The upper organic layer was separated and transferred into a separate tube and evaporated under a nitrogen stream at 40 °C. The residue was reconstituted in 100 µL of methanol, and 1 µL was injected into the GC-MS.

The procedure of extraction from urine: To 4 mL of urine sample, 1 mL of diphenylamine 0.01% in methanol was added. The samples were alkalinized using 2M kalium hydroxide (up to pH 10). Ten milliliters of the solvent mixture (n-hexane–2-propanol, 97:3) was added and then vortexed for 15 min, and then the samples were centrifuged for 10 min at 3400 rpm and 15 °C. The upper organic layer was separated and transferred into a separate tube and evaporated under a nitrogen stream at 40 °C. The residue was reconstituted in 100 µL of methanol, and 1 µL was injected into the GC-MS.

### 4.7. Instrumentation

A Thermo Electron Corporation Focus gas chromatograph coupled to a DSQII mass spectrometer operating with quadrupole and electron ionization (EI) ion source was used. A TR-5MS (stationary phase 5% phenyl polysilphenylene siloxane) capillary column (30 m × 0.25 mm × 0.25 µm film thickness) was used for the analysis of samples. Specialized software Xcalibur ver. 1.2 with the NIST 02 mass spectral library was used for data collection and processing.

### 4.8. Chromatographic Separation and Mass Spectrometric Detection Conditions

The determination of methadone was performed under the following temperature gradient program: an initial temperature of 150 °C was held constant for 1 min; then, it was increased to 220 °C at a flow rate of 10 °C/min and then increased to 280 °C at a flow rate of 30 °C/min. The temperature was held constant for 1 min. The total time of analysis was 12 min. The carrier gas (helium) flow rate was set at 1 mL/min.

Mass spectrometric conditions: Solvent elution time: 6 min.; mass range: 50–650 u.a.m.; injector temperature: 220 °C; work in split mode; transfer line temperature: 260 °C; ionization source temperature: 200 °C.

## 5. Conclusions

A validated procedure for the GC-MS determination of methadone in urine using LLE has been applied, discussed, and compared with the determination of methadone in plasma. A comparison of the procedure between the two types of biological samples has revealed that urine provides higher methadone levels than plasma, revealing a wider distribution of the analyte in urine than in plasma. Moreover, for one of the patients under methadone treatment, the plasma analysis provided a negative result. A disadvantage of plasma detection could be the lower available volume of samples. In addition, the complete sample preparation for urine requires less time than that for plasma, as blood samples need to first be collected in an appropriate type of vacutainer and need to be centrifuged and separated prior to being processed. Therefore, the proposed procedure can be used in clinical laboratories for the rapid determination of methadone levels in urine rather than in plasma. This procedure can be also applied for the monitoring of methadone substitution treatment.

## Figures and Tables

**Table 1:** Various methods for methadone and EDDP determination in biological samples.

Matrix	Analyte	Internal Standard	Method of Extraction	Method of Analysis	Reference
Cadaveric blood	MTD EDDP	No IS	LLE Organic mixture solvents at pH 9.0	GC-MS	[6]
Plasma Dried blood spots (DBS)	MTD LOQ 0.05 ng/mL (plasma): 1 ng/mL (DBS); EDDP LOQ 0.025 ng/mL (plasma): 0.5 ng/mL (DBS)	Diphenhydramine	LLE, Ethyl acetate	HPLC-MS/MS, Acetonitrile (gradient from 10–34%) in 0.1% formic acid (pH 6.5), Chiral-AGP 150 × 4.6 mm, 5 μm	[12]
Hair Skin	d,l-MTD LOQ 25 µg/L (skin) LOQ of 50 pg/mg (hair); EDDP LOQ 25 µg/L (skin) LOQ of 50 pg/mg (hair)	d,l-Methadone-D9 EDDP-D3	SPE, Chromabond HR-XC	GC-MS/MS (hair), LC-MS/MS (serum and sweat patches), Water with 0.1% formic acid and acetonitrile with 0.1% formic acid, RP 18 EC 150 × 2.0 mm, 2.7 µm	[13]
Femoral venous blood Brain (medulla)	MTD LOQ 0.01 mg/L	Methadone-D9	SPE, Bond Elut Certify	GC-MS	[10]
Whole blood	MTD LOQ 25 ng/mL	No IS	SPE, Bond Elut Nexus, Supelco ENVI Florisil, Supelco LC-18, Amberlite XAD2, LLE, Ethyl acetate–dichloromethane 1:3 *v*/*v*	GC-MS	[14]
Urine	MTD EDDP LOQ: 25 ng/mL	Not mentioned	Not mentioned	HPLC-MS/MS, Water/acetonitrile (20:80 *v*/*v*) with 1% formic acid, C18 100 × 3.2 mm, 3 µm	[15]
Plasma	MTD EDDP	Diphenylamine	LLE, Hexane–2-propanol 97:3, *v*/*v*, pH 9	GC-MS	[9]
Urine	EDDP LOQ 40 ng/mL	EDDP-D3	LLE, Chloroform–2-propanol 90:10, *v*/*v*	GC-MS	[16]
Plasma	MTD LOQ 0.5 ng/mL	Diphenylamine	LLE, Tertbutyl methyl ether	GC-MS	[17]

**Table 2:** Demographic and toxicological characterization of the study group.

Parameter	Group Characteristics	
Total cases	14	
Sex	14.29% females (2); 85.71% males (12)	
Male/female ratio	6	
Age (years) (mean ± SD)	32.23 ± 9.90 (range 19–47 years) Males 32.73 ± 10.71 Females 29.5 ± 3.54	
Frequency of age groups	<20 years 7.14% (1/14); 20–30 years 42.86% (6/14); 30–40 years 21.43% (3/14); >40 years 28.57% (4/14)	
History of opiate use	78.57% (11/14)	
MMT history - Pearson correlation of age and MMT history	50% (7/14) R = 0.8822, *p*-value is 0.00003	
Methadone dose (mg/day) (mean ± SD)	95.71 ± 20.70 (range 50–110)	
Associated medication	% (no. of reports/total reports)	
	Antidepressants (AD)	Escitalopram 7.14 (1/14) Trazodone 14.29 (2/14) Mirtazepine 28.57 (4/14)
	Anxiolytics/hypnotics (AX)	Cinolazepam 7.14 (1/14) Diazepam 28.57 (4/14) Zolpidem 14.29 (2/14)
	Antipsychotics (AP)	Risperidone 21.43 (3/14) Haloperidol 14.29 (2/14) Quetiapine 14.29 (2/14) Flupentixol 7.14 (1/14) Olanzapine 21.43 (3/14) Levomepromazine 7.14 (1/14)
	Anticonvulsants (AC)	Valproic acid 57.15 (8/14) Carbamazepine 28.57 (4/14) Gabapentine 42.86 (6/14) Levetiracetam 7.14 (1/14)
Pattern of treatment (drug, %, count/total count)	MTD 14. 29 (2/14) MTD + AD + AX + AP + AC 35.71 (5/14) MTD + AD + AC (7.14 (1/14) MTD + AC + AP 14.29 (2/14) MTD + AP + AC + AX 14.29 (2/14) MTD + AC + AD + AX 7.14 (1/14) AC + AD 7.14 (1/14)	

**Table 3:** Determination of methadone levels in urine and plasma.

No.	MTD (mg)	U	P	q-MTD m/z = 72 t_R_ = 10.64	q-EDDP m/z = 277 t_R_ = 9.92	Q-Conc. MTD (µg/mL)
1	−	Yes	−	−	−	−
2	100	Yes	−	+	−	0.072
3	−	Yes	−	−	−	−
4	100	Yes	−	+	−	1.06
5	100	Yes	−	+	+	4.336
6	110	Yes	−	+	+	3.012
7	110	Yes	−	+	+	6.697
8	−	Yes	−	−	−	−
9	−	−	Yes	−	−	−
10	−	−	Yes	−	−	−
11	−	−	Yes	−	−	−
12	50	−	Yes	−	−	−
13	−	−	Yes	−	−	−
14	100	−	Yes	+	−	0.013

No. (patient number), MTD (methadone oral dose), U (available urine sample), P (available plasma sample), EDDP (2-ethylidene-1,5-dimethyl-3,3-diphenyl pyrrolidine), “−” (missing sample or negative response), “+” (positive result); q (qualitative analysis), Q (quantitative analysis).

## Data Availability

The data presented in this study are available on request from the corresponding author.
